# When the clinician becomes the manager: safety planning as intrapsychic enactment and a testable Internal Family Systems reframing of suicide-specific care

**DOI:** 10.3389/fpsyt.2026.1903433

**Published:** 2026-10-15

**Authors:** Jörg Strobel

**Affiliations:** 1 College of Medicine and Public Health, Flinders University, Adelaide, SA, Australia; 2 Mental Health Directorate, Barossa Hills Fleurieu Local Health Network, Glenside, SA, Australia

**Keywords:** collaborative care, concealment, enactment, iatrogenesis, Internal Family Systems, nondisclosure, safety planning, suicide prevention

## Abstract

Safety planning depends on disclosure. Yet many suicidal patients conceal their suicidal intent from clinicians, often fearing the consequences of disclosure. Critics of contemporary suicide-prevention frameworks increasingly warn that a detection-and-containment approach may foster a defensive, algorithmic practice, but an encounter-level account of how such an approach may become counter-therapeutic has remained underdeveloped. Drawing on Internal Family Systems (IFS) theory, we propose one such account. We argue that suicidality is often best understood as a protective response to unbearable distress, conceptualized within IFS as a “firefighter” stance: a protective action whose catastrophic outcome nonetheless serves a function. More novelly, we suggest that containment-oriented safety planning can recruit the clinician into the `manager' role within the patient’s internal system, recreating within the therapeutic dyad the very manager–firefighter polarity that may contribute to the crisis. From this follows a specific paradox: a system that depends on disclosure may, under certain conditions, suppress the very disclosure on which it relies. The contribution of this paper is neither the functional interpretation of suicidality nor the critique of containment-oriented care, both of which are well established in the literature. Rather, it is the proposal that the clinical encounter itself may operate as an enactment of the patient’s own manager–firefighter polarity, and that it may create a concealment mechanism through which suppression-oriented care may undermine its own aims. From this account, we derive a falsifiable prediction that distinguishes it from alliance- and autonomy-based explanations: a containment-oriented approach should produce selective concealment of suicidal intent despite preserved engagement—a dissociation between sustained alliance and concealed suicidal intent that those explanations do not predict. Conditions for disconfirmation are specified.

## 1 Introduction

The Safety Planning Intervention (SPI; 1) has become, in little more than a decade, the closest thing suicide prevention has to a universal clinical procedure. Its six collaboratively constructed components—recognizing warning signs, internal coping strategies, social contacts for distraction, people to ask for help, professionals and crisis services, and reducing access to lethal means—are now taught across emergency, inpatient, and community settings, and in adults presenting to emergency departments the intervention is comparable to usual care (2). In parallel, the Zero Suicide Framework (ZSF) and its derivatives have elevated suicide-specific care from a clinical task to an organizing principle of mental health–system design, with the aspiration that suicide deaths among people receiving care can be reduced to zero (3).

Alongside this growing adoption, concerns have emerged regarding the unintended consequences of approaches organized primarily around suicide-risk detection and containment. Recent critiques argue that such systems may shift towards defensive, algorithmic forms of practice in which the procedural management of risk displaces deeper psychological understanding (4). Yet these critiques largely remain descriptive. They identify a potential problem without specifying the encounter-level mechanism through which a containment-oriented stance may become counter-therapeutic. Why might an intervention that depends upon disclosure inadvertently suppress it? The present paper proposes an answer.

Drawing on Internal Family Systems (IFS) therapy (5, 6), we suggest that containment-oriented safety planning can, under certain conditions, recruit the clinician into the manager role of the patient’s own internal system. Rather than resolving the manager–firefighter polarity associated with suicidal crises, the clinical encounter may inadvertently reproduce it. From this account follows a specific paradox: a system that depends on disclosure may, under certain conditions, suppress the very disclosure upon which it relies.

It is useful to separate three claims at the outset, because they carry very different meanings.

- Claim A: suicidality is frequently “functional” and “protective” rather than simply a symptom to be suppressed. This claim is well established and is shared across the Aeschi narrative-action tradition (7), the functional analysis embedded within dialectical behavior therapy (8), and the IFS literature itself (9). We attribute it; we do not claim it.
- Claim B: a medicalized detection-and-containment paradigm can become impersonal, autonomy-eroding, and iatrogenic. This too is established as critique, from the Aeschi group through to contemporary analyses of Zero Suicide framework (4). We build upon it.
- Claim C: conventional safety planning, as enacted, may recapitulate the patient’s own manager–firefighter polarity, with the clinician recruited into the manager role. To our knowledge, this dynamic has not been previously articulated. It is the principal contribution of this paper and the basis of the testable prediction developed here.

The argument proceeds as follows. We first describe the manager enactment (Section 2) and the IFS conceptualization of suicidality on which it depends (Section 3). We then position the enactment thesis in contrast to related frameworks (Section 4) and specify the concealment mechanism that gives it clinical and empirical significance (Section 5). Section 6 offers a reframing of safety planning that preserves its protective functions while changing the clinician’s stance. Section 7 illustrates the same logic at the system level through the example of South Australia’s *Towards Zero Suicide* initiative. Section 8 states the central hypothesis in falsifiable form, together with its discriminating prediction and conditions for disconfirmation. Section 9 addresses objections and limitations. Section 10 concludes.

One clarification is essential before proceeding, as the argument is easily misread. This paper does not argue against safety planning, risk assessment, or protective action of any kind; it does not suggest that any patient be denied a safety plan, that means restriction be relaxed, or that acute danger be met with anything less than the full measure of protection it demands. In particular, means restriction has the strongest evidence and the clearest mechanism of any element discussed here, and nothing in what follows weakens it. The claim is narrower, and entirely about manner: that the stance with which protective measures are delivered is itself clinically active, and that a suppressive, done-to-the-patient delivery may, for some patients, erode the very disclosure on which those measures depend. Every reframing we propose retains each protective component; it changes only how, and in what sequence, the components are offered.

## 2 The problem: safety planning as a manager’s enactment

Four terms recur throughout what follows and are worth distinguishing before the argument proceeds. First, safety planning denotes the intervention itself—the collaboratively constructed artefact of warning signs, coping strategies, contacts, and means restriction (1). Second, containment denotes a stance or mode of delivery: a clinician’s orientation towards controlling, pre-empting, or suppressing a feared outcome, which may or may not accompany safety planning and is not synonymous with it. Third, coercion denotes the patient’s subjective experience of that stance—a quality of the encounter, not a feature of any specific intervention, and one that the same protective action can produce or avoid depending on how connection and control are preserved (Section 5). Fourth, risk management denotes the institutional and documentation layer within which both occur—the procedural, medico-legal apparatus that can operate independently of, and sometimes in tension with, the clinical encounter itself. The claim developed here concerns containment as a stance, not safety planning as intervention; the two are not interchangeable, and the argument does not target the latter.

The difficulty is not the safety plan but the stance with which it is most often used. Under the time pressure of an emergency department or an acute ward, the plan is frequently completed “to” the patient rather than “with” them—a procedure to be discharged, a piece of risk-management documentation, a box to be ticked before disposition. Under such conditions, the suicidal part may experience the interaction as “stop doing this; here are the techniques by which you will be made to stop, and here is how your access to the means of stopping will be reduced”. The clinician identifies a dangerous impulse and mobilizes strategies to control, distract from, and override it.

In the language of IFS, this is the behavior of a “manager”: a proactive, controlling protector whose aim is to prevent a feared inner state from surfacing through pre-emption and suppression (6). We propose that when a clinician engages with a suicidal patient in this manner, the therapeutic dyad replicates the patient’s own internal system. The patient’s system already contains a manager (the part that has spent a lifetime trying to keep unbearable affect out of awareness) locked in a polarity with a firefighter (the part that acts, catastrophically, once the manager's defences fail). Containment-mode safety planning installs the clinician, and behind the clinician the whole apparatus of risk management, as a second, external manager — lined up on the same side of the same polarity. The crisis is managed; nothing is understood. The firefighter, far from being engaged, is now opposed by one more party.

This constitutes a second-order observation in the cybernetic sense: the clinician is not a neutral observer detached from the patient’s difficulties but a participant whose stance becomes part of the system they seek to influence (10). In this account, that participation takes a specific form: the clinician may be recruited into the manager pole of the patient’s own manager–firefighter polarity.

The phenomenon is not unique to IFS. Enactment is an established concept in relational–psychoanalytic literature, where it refers to the clinician’s unconscious recruitment into a role predetermined by the patient’s internal world (11, 12). What this literature adds, and what is directly relevant here, is the observation that enactment is often driven not by the clinician’s lapses but by their adherence to standard technique: rigidly following procedure can be the very means through which the patient’s expectations are played out rather than examined. This paper can therefore be interpreted in two ways—as an IFS-specific claim about the manager pole, and, more broadly, as the proposition that a manualized, containment-oriented safety-planning procedure is a structurally probable site of enactment, with IFS providing the content of the role into which the clinician is recruited.

The consequences of misinterpretation are not merely theoretical. Approximately half of individuals experiencing suicidal ideation deny it when asked directly, and approximately half of those who die by suicide had denied suicidal ideation in the week or month prior to their death (13). Denial of ideation is, in itself, an insufficient indicator of risk; nondisclosure is not a peripheral nuisance but the central obstacle to the entire detection-and-management effort. If the safety-planning approach influences disclosure—and we will argue that it does—then the clinician’s stance is not superficial. It is clinically active and potentially decisive.

In brief, and stated schematically before the detailed argument unfolds, a containment-oriented stance risks recruiting the clinician into a manager role (Section 2), which produces protective concealment (Section 5), with consequences for clinical outcomes that a reframed stance (Section 6) is designed to avert.

## 3 Suicidality as a firefighter stance (an attributed reading)

IFS organizes the inner system into three functional classes of vulnerable and protective parts (5, 6). Exiles are young, wounded parts that carry the burdens of shame, terror, and worthlessness acquired, often early, in conditions of relational injury. Managers are protective parts that run daily life proactively, seeking security through control and the pre-emptive exclusion of exiles from awareness. Firefighters are also protective parts and are the system’s emergency service: when an exile is activated and breaks through the managers’ defenses, firefighters act—urgently, indiscriminately, and without regard to cost—to extinguish the unbearable affect. Their repertoire is the familiar catalogue of self-destruction: substance use, bingeing, dissociation, self-harm, and, at the extreme, suicidal action.

Within this framework, the suicidal part is not the pathology; it is a protector whose protective logic has reached its terminus. Its communication, when it is listened to, is some version of “the pain is unsurvivable, and I can guarantee it will stop”. Suicide functions here as the ultimate firefighter maneuver—the one intervention that promises, with total reliability, to end the activation of the exile. The lethality is downstream of a protective intention.

This reading is not new, and intellectual honesty requires emphasizing that it is not the contribution of this paper. Sweezy (9), the closest peer-reviewed IFS treatment to the topic, sets out precisely this taxonomy, naming suicide among the dangerous behaviors that firefighters may urge while insisting that such parts remain protective in intent. The broader IFS literature treats chronic suicidality in these terms (6). Two consequences follow. First, the meaningful clinical question shifts from “how do we stop this behavior?” to “what does this part believe is unsurvivable, and what is it protecting?” Second, the suicidal part is recognized as standing in “polarity” or in a polarized relationship with the system’s managers, such that greater emphasis on suppression tends to escalate, not soothe, the firefighter. It is the second consequence, turned outward onto the clinician, that the rest of this paper develops.

## 4 Prior art: what has been claimed and what remains

The enactment thesis earns its place only within a well-mapped field. Five bodies of work come close to this thesis, and the precise ways in which each falls short of it define the contribution of this paper.

### 4.1 The Aeschi tradition and ASSIP

Michel, Valach, and colleagues developed a program on a critique of the traditional risk paradigm, which they viewed as overly medicalized and impersonal. They proposed a positive alternative rooted in a theory of suicide as goal-directed action, incorporating a narrative interview and a shared, collaborative understanding of the patient’s suicidality. A core principle of their work is curiosity about the “function” of suicidal urges (7, 14). This approach was operationalized and tested as the Attempted Suicide Short Intervention Program (ASSIP), which is supported by randomized-trial evidence (15). The Aeschi tradition thus encompasses Claim A and much of Claim B. However, its concept of “function” refers to the goal an action serves within a life narrative, not a protective subpersonality in a polarized internal system. Furthermore, it lacks a concept of the clinician unconsciously enacting a complementary internal role, as action theory does not generate this idea. It prompts the clinician to be curious about function but does not analyze why standard procedures structurally impede that curiosity.

### 4.2 The collaborative assessment and management of suicidality (CAMS)

CAMS (16) is a theoretically agnostic, suicide-focused framework structured around the patient’s idiosyncratic drivers of suicide. These drivers are assessed collaboratively, with the patient positioned as co-author of the plan. The aim is to shift drivers rather than eliminate them, so that the patient no longer feels they have to die. It has a substantial evidence base from trials. CAMS’s theoretical agnosticism is precisely the gap the present account addresses: it asks “what drives this?” However, it is deliberately silent on “what a driver is” and “how to relate to it”. IFS provides one answer—a protector within a polarity, to be engaged from a specific stance. We therefore propose IFS not as a rival to CAMS but as a theory of the driver that can be integrated within it. This represents the safest and strongest position available, as it operates within an evidence-based framework rather than displacing evidence-based practice.

### 4.3 Dialectical behavior therapy

Dialectical behavior therapy (DBT) is the approach most likely to be confused with the present argument because it, too, understands suicidal and self-harming behavior as functional rather than meaningless or irrational. Within DBT, suicidality is conceptualized as a behavioral strategy for regulating overwhelming affect and is addressed through validation, behavioral analysis, and skills development (8). The key distinction lies not in the function attributed to suicidality but in the account of the therapeutic relationship: DBT explains the function of the behavior but does not include a model of therapist parts or intrapsychic polarities and therefore cannot readily generate an enactment analysis. The present paper proposes that containment-oriented care may recruit the clinician into a complementary role within the patient’s existing manager–firefighter conflict. Notably, Sweezy (9), writing from a DBT background, suggested a complementary relationship between containment and IFS-informed depth work. The present paper accepts that sequencing while suggesting that the interface between the two approaches may reveal an overlooked concealment mechanism.

### 4.4 The policy critique

Recent critiques of the Zero Suicide framework argue that systems organized around suicide-risk detection may gravitate towards defensive, algorithmic forms of practice, prioritizing documentation and risk management over comprehensive formulation (4). This observation is highly relevant to the present argument as it identifies a phenomenon many clinicians will recognize. The critique is principally concerned with systems-level dynamics, however, rather than encounter-level mechanisms. The present paper can therefore be read as complementary to this work: if defensive practice is the phenomenon, manager enactment and protective concealment are proposed as one mechanism through which it may become clinically consequential. On this account, containment-oriented care may inadvertently suppress the very disclosure upon which detection depends.

### 4.5 Self-determination theory and motivational interviewing

Self-determination theory or SDT (17) and motivational interviewing (MI) (18) offer the most direct account of why an autonomy-supportive, non-directive stance improves engagement: autonomy-thwarting delivery frustrates a basic psychological need, and ambivalence resolves through evocation rather than confrontation. These frameworks are, in fact, the natural source of the atheoretical measures used in Section 8 to operationalize clinician stance without presupposing IFS. What they do not supply is a model of the clinician’s participation in the patient’s internal conflict: SDT explains why autonomy support works, and MI provides techniques for offering it, but neither generates the claim that a directive stance recruits the clinician into a complementary role within a pre-existing intrapsychic polarity. The present account is compatible with, and indeed borrows the measurement apparatus of, both frameworks; its distinct contribution is the enactment mechanism that neither framework explicitly describes.

The negative space is now precisely defined. Existing collaborative and functional approaches have established that suicidal behavior is goal-directed and protective and that a containment-oriented stance can be counter-therapeutic; IFS has, in parallel, reframed the suicidal impulse as a protective firefighter. What none has articulated is an account of the standard safety-planning encounter itself as an intrapsychic enactment—the clinician recruited into the manager pole of the patient’s own system—together with the mechanism by which that enactment defeats its own purpose.

## 5 The mechanism: why suppression backfires

If suicidality is a firefighter locked in a polarity with managers, and if containment-mode safety planning installs the clinician as a second manager, then three specific and clinically consequential risks follow.

### 5.1 Polarization

Pressing on one pole of an internal polarity characteristically strengthens the other. A safety plan delivered as an instruction to desist can intensify the very part it targets, because that part now faces not only the original distress but the additional threat of being shut down before it has fulfilled its protective function. Escalation under coercion is not evidence that firmer suppression is required; it is often evidence that the suppression is feeding the polarity. If the present account is correct, the consequence may, under some conditions, include a paradoxical short-term intensification of the suicidal urge itself, not merely reduced cooperation. Because it follows so directly from the polarity model, this is also the most theory-specific and most vulnerable element of the account; the discriminating weight of the argument rests not here but on the concealment described next.

### 5.2 Concealment

This is the most under-appreciated and potentially the most dangerous, and it is where the account becomes empirically tractable. A firefighter who experiences the clinical environment as hostile to its existence will learn to operate covertly. The patient discovers that disclosure leads to control—to admission, surveillance, the removal of autonomy—and reasonably concludes that the route to preserving the part’s options is to stop talking about it. Crucially, this concealment need not present as disengagement: the patient may remain in treatment, complete the plan, and appear cooperative while withholding suicidal intent—concealment masked by engagement. This is not a theoretical worry. In the one study that asked psychotherapy clients directly, 70% of those who concealed suicidal ideation cited fear of its practical consequences outside therapy—chiefly involuntary hospitalization—and nearly half said they would be more forthcoming if that threat were reduced (19); shame is a further documented motive. Concealment is, in substantial part, a response to anticipated coercion. It follows that suppression-mode safety planning can purchase the appearance of safety—a completed plan, a reassuring denial—at the cost of the disclosure on which real safety depends. This is the precise mechanism by which a well-documented plan and a confident denial can precede a fatal act.

### 5.3 Rupture

A patient whose suicidal part is met primarily as a problem to be eliminated rather than as a protector to be understood may experience the encounter as an attunement failure. For people whose distress already centers on being unseen or misunderstood, such interactions may reinforce the very meanings that contribute to the crisis.

These are not only theoretical risks; a body of qualitative and lived-experience research now documents both the concealment and its motives. Across populations—psychotherapy clients, primary-care and emergency patients, young people, and veterans—a large fraction of those experiencing suicidal ideation report withholding it from clinicians (a community sample of suicidal youth found that 39% had never disclosed to their clinician; an adult inpatient sample, 51.5%), and disclosure is associated with the strength of the therapeutic alliance (20, 21). When patients are asked why, the same reasons recur—fear of stigma, fear of the clinician’s overreaction, and fear of losing autonomy through hospitalization—while what they identify as enabling disclosure is nonjudgemental listening and care without overreaction (22). This is the concealment loop in patients’ own words: disclosure is titrated against the anticipated coercive response, exactly as the enactment account predicts.

Honesty—and the avoidance of an echo chamber—requires acknowledging the other half of this literature, which also bears on the boundary conditions of our hypothesis: we specify in Section 8 that the predicted effect should be stronger in post-acute than acute settings, larger among patients with a prior history of involuntary admission, and stronger in adolescents than adults, for whom autonomy concerns are especially salient. The same qualitative tradition finds that protection and containment are frequently experienced as safety rather than coercion, but conditionally. In a systematic review of suicidal inpatients’ experiences, patients felt safe when three needs were met—connection, protection, and control—and felt unsafe, with escalating suicidal behavior, when they were not (23); a companion interview study found safe practice was experienced as recognition of suicidality, receiving tailored treatment, and being protected by adaptive rather than standardized practice (24). The lesson is not that containment is iatrogenic in itself—plainly, it is not—but that its manner determines whether it is metabolised as protection or as coercion: protection offered with connection and a preserved sense of control is experienced as safety, while protection imposed without them is experienced as an enactment of the manager role. This is precisely the variable our hypothesis isolates, and it disciplines the claim—not that every directive act harms, but that stance, not structure, is decisive.

The concealment risk closes a loop that is, we suggest, the heart of the matter. A detection-and-accountability system depends on patients disclosing enough information to be identified as at-risk-in-care. However, the more forcefully such a system pursues detection and containment, the more it may activate the protective concealment that the nondisclosure literature documents—so that the system can suppress the very signal it is built to detect. At the level of individual encounters, this is the manager’s enactment producing the outcome it most fears. At the system level, it is the mechanism underlying the “defensive, algorithmic practice” that Ouliaris et al. (4) describe but do not explain. None of this argues against protective action. It is an argument that the manner of protective action is itself clinically active—that the stance taken is a variable, not a constant.

## 6 The reframe: IFS-informed safety planning

What follows is a reframing, not a replacement. It retains every protective function of conventional planning and changes the stance and the sequence in which they are deployed.

### 6.1 Stabilization before depth

IFS practice unequivocally states that work with the most vulnerable parts is contraindicated until there is sufficient stability and the consent of protectors; one does not open a wound a system cannot yet hold (6). Translated to the suicidal context, this yields a phase distinction that resolves much of the apparent tension between understanding the suicidal impulse and keeping the person alive.

In the acute phase, with imminent risk, the priority remains stabilization and protection—including means safety, increased support, and, where genuinely indicated, a higher level of care. This is not the moment for relational depth work. The IFS contribution at this phase is principally one of stance: protective steps are negotiated “with” the suicidal protector rather than imposed against it. The aim is not to abandon containment but to avoid reproducing the manager–firefighter dynamic described in Section 5. Only in a subsequent phase, once acute danger has receded, does the relational work proper begin: building a more regulated and less defensive stance towards the suicidal impulse, getting to know the protector, and, in time and with consent, witnessing the pain it has been attempting to manage.

This distinction also answers the objection implicit in the sequencing model of Sweezy (9). Our critique is not of staged containment nor of structure having a legitimate role; Sweezy’s Stage 1/Stage 2 formulation remains entirely compatible with the present account. The manager’s enactment arises when containment is delivered as suppression—without a decoding function and without awareness of stance—and is mistaken for the whole task rather than its first phase.

### 6.2 Self, not manager

The single most important variable is the clinician’s stance. IFS describes “Self” as a quality of presence characterized by curiosity, calm, compassion, clarity, courage, confidence, and connectedness (6). Whether Self is understood as an ontological reality or simply as a regulated, non-defensive therapeutic stance matters less than the clinical consequences that follow from it. A clinician planning for safety from this stance does not primarily encounter a risk to be managed; they encounter a frightened and overburdened protector that has come to believe catastrophe is the only reliable solution. The task becomes understanding before intervening.

This is not unique to IFS. It closely resembles the collaborative stance described within CAMS, where the clinician seeks to understand the suicidal struggle from the patient’s perspective and positions themselves as an ally rather than an adversary (16). The significance of the stance is practical rather than philosophical: a “firefighter” met with curiosity rather than coercion has less reason to conceal, less reason to escalate, and a reason to remain in dialogue. In the terms developed in Section 5, the stance addresses the mechanisms of polarization, concealment, and rupture at their source.

### 6.3 The components, reframed

The familiar safety-plan elements survive intact; their meaning changes.

- Warning signs: Rather than functioning solely as a checklist of triggers, warning signs become opportunities to trace the sequence through which the crisis develops: which vulnerability has been activated, what protective response emerged, and at what point does the “firefighter” take over. The aim is to move from “I want to die” towards “a part of me is becoming overwhelmed, and another part is preparing to act”—a shift that creates distance from the impulse without dismissing it.
- Internal coping strategies become practices that help the patient develop enough perspective to remain present to the suicidal part rather than being overwhelmed by it. The aim is not distraction from the “firefighter” but enough separation from it that it is no longer carrying the burden alone.
- Social supports and crisis contacts remain essential, but their function changes subtly. Rather than identifying people who will stop the patient, the emphasis becomes identifying people who can help them remain connected and regulated when distress escalates. The question shifts from “who will stop me?” to “who can be with this part of me?”
- Means safety remains unchanged in principle and is retained in full; it is the component with the strongest evidence base and the clearest mechanism. What changes is how it is understood. The clinician makes the rationale explicit and shared: the firefighter’s job is to ensure the pain can be ended if it becomes unbearable; the clinician’s job is to ensure the person is not forced into something irreversible in a moment that will pass; reducing access buys the whole system time without removing the protector’s role. Framed this way, means safety becomes an act of alliance with the “firefighter’s” protective intention rather than a restriction imposed against it. Nothing in the present account weakens the rationale for lethal-means counselling; if anything, it strengthens it.

Finally, the formulation itself should record the function of the suicidal part not merely its presence and severity: “Chronic suicidal ideation, denies current intent” is clinically inert. Suicidal protector activated by experiences of humiliation; believes death is the only reliable end to shame; currently in dialogue; has agreed to means safety as time-buying" is a working object the next clinician can use — and, we note, a more defensible medico-legal artefact, because it evidences reasoning rather than mere detection (**Table 1**).

**Table 1:** The same safety-plan components delivered in two stances.

Component	Standard (containment) delivery	IFS-informed (self-led) delivery
Warning signs	A checklist of triggers to watch for and avoid.	A traced sequence—which part is activated, by what, and when the firefighter takes over; “a part of me is starting to give up.”
Means safety	Access is removed; the step is documented for risk management.	A transparent agreement with the protector: reducing access buys the system time without removing the firefighter’s role.
Formulation	“Chronic suicidal ideation, denies current intent.”	“Suicidal protector activated in response to humiliation; believes death is the only reliable end to shame; in dialogue; agreed to means safety as a way of buying time.”

The content is largely unchanged; the relationship to the suicidal part is not.

A composite illustration: The following vignette is composite, drawn from no single case, and does not describe an identifiable patient.

A patient presents to an emergency department after a period of escalating distress, endorsing passive suicidal ideation but denying current intent. Following standard protocol, the treating clinician moves briskly through the six SPI components, framing each as a means of preventing the patient from acting: warning signs are elicited as a checklist of triggers to avoid; means restriction is presented as a condition of discharge; crisis contacts are recorded as people who will intervene should ideation return. The patient nods, agrees, and signs the plan. Nothing in the encounter is false, and nothing in the plan is wrong. However, the patient’s own account, given days later under different conditions, is that the encounter felt like being managed—monitored and quietly appraised for compliance—and that suicidal planning already underway went undisclosed, because disclosure would have meant admission. The plan was completed; the disclosure it depended on was not.

A second clinician, working from the one described in Section 6, opens differently—not “let’s make sure you’re safe,” but “tell me what part of you needed this to be an option”. The same six components are covered, with means safety addressed directly and without equivocation, but each is arrived at through the patient’s own account of what the suicidal part is protecting against, rather than imposed as a precondition for discharge. The resulting plan looks similar on paper to the first. What differs is what the patient discloses under it, and to whom.

## 7 The paradigm at scale: South Australia as an illustrative case

South Australia’s Towards Zero Suicide (TZS) initiative provides a useful illustration of the broader detection-and-containment paradigm that now shapes much of contemporary suicide prevention (25). Like other implementations of the Zero Suicide Framework (developed from the Henry Ford “Perfect Depression Care” initiative; 26), TZS seeks to reduce suicide through earlier identification of risk, standardized suicide-specific care, enhanced monitoring, and improved continuity of treatment. The initiative addresses real and important problems, including fragmented care and the high-risk period following discharge.

Viewed through the lens proposed here, such frameworks can be understood as organisational analogues of the clinician's manager. Their central tasks are anticipation, detection, monitoring, standardization, and prevention of a feared outcome. The comparison is not intended to pathologize these systems: within IFS, managers are protective parts whose efforts are often indispensable. The point is not that control, monitoring, or standardization are inherently harmful but that systems organized around preventing a feared outcome may become vulnerable to the same tensions that emerge within individuals.

If concealment is partly a response to anticipated coercion, as the disclosure literature reviewed earlier suggests, then a system organized primarily around detection and containment may encounter a parallel paradox. Efforts to identify and manage suicide risk may, under some conditions, increase incentives to withhold information about suicidal intent. On this account, the challenge is not detection itself but the possibility that detection and disclosure can be in tension. This perspective offers a possible encounter-level mechanism underlying critiques that detection-and-containment systems may drift towards defensive, algorithmic practice (4): if containment-oriented interactions promote protective concealment, then a system may inadvertently suppress the very signal upon which its detection strategy depends.

## 8 The hypotheses

We now state the central empirical claim in falsifiable form. Although the present account generates several predictions, only one is genuinely discriminating. H1 places the theory at risk.

H1 (the discriminating prediction): Containment-oriented delivery produces “selective concealment of suicidal intent despite preserved engagement”. The patient remains in treatment, completes the safety plan, and presents as cooperative, while selectively withholding information—current intent, access to means, the operative plan—that would reduce their suicidal options. It is the dissociation that is discriminating: engagement and alliance are maintained, or even appear enhanced, while the one signal on which safety planning depends is the signal withheld. On the enactment account, this is precisely what the firefighter would do—keep the clinician reassured in order to keep the option intact.

What distinguishes this prediction from its rivals is the dissociation it specifies. Alliance-, autonomy-, and common-factors accounts all predict that a coercive, controlling delivery degrades engagement and disclosure together; none predicts that a patient who remains engaged, allied, and outwardly cooperative will selectively withhold intent. The account therefore predicts a particular pattern rather than a mere main effect: concealment of intent that is uncoupled from, or even inversely related to, observable disengagement.

Secondary predictions: Two further predictions follow from the account but do not, on their own, distinguish it.

H2 (disclosure): Holding safety-plan content constant, a more autonomy-supportive, curiosity-driven, non-coercive delivery of safety planning increases the patient’s disclosure of suicidal ideation and intent relative to a more directive, containment-oriented delivery.

H3 (mediation): The effect of delivery style on subsequent engagement and short-term outcomes is mediated by disclosure and therapeutic alliance.

H2 and H3 are not, in any fundamental sense, unique to the present account; similar predictions follow from CAMS, motivational interviewing, self-determination theory, and common-factors models of psychotherapy. Positive findings would be consistent with the present theory but would not distinguish it from several plausible alternatives. H1 is the prediction that places the theory at risk.

An auxiliary prediction: The polarity model generates one further prediction—that containment-oriented delivery may produce a paradoxical short-term intensification of suicidal urges, not merely a failure to reduce them. Because this prediction follows directly from IFS-specific assumptions, this is the most theory-dependent and therefore the most vulnerable element of the account. We therefore treat it as corroborating rather than discriminating, and do not make it central to the test: its presence would strengthen the mechanistic reading, but its absence would not, by itself, defeat the concealment claim on which H1 turns.

Operationalizing stance: To avoid circularity, the central independent variable—clinician stance—should be measured using constructs that do not presuppose IFS: autonomy support versus control, collaborative versus directive framing, and curiosity about function versus emphasis on control and risk management. Existing observational frameworks from motivational-interviewing fidelity coding and CAMS adherence research provide natural starting points, and perceived autonomy support can be captured from the patient’s perspective with an instrument such as the Health Care Climate Questionnaire. The value of such measures is precisely that they can test the theory without assuming it. The downstream variables likewise have established measures: engagement and therapeutic alliance via attendance and retention and the Working Alliance Inventory or a session-rating measure—so that preserved engagement can be documented—and, for the auxiliary urge prediction, short-term suicidal urge via the Suicide Crisis Inventory [the construct whose link to differential clinician-versus-researcher disclosure Rogers et al. (27) demonstrated].

Measuring concealment: The obvious challenge is that concealed intent is, by definition, hidden. It can nonetheless be operationalized through a disclosure discrepancy: adult psychiatric patients disclose suicidal intent more readily to researchers than to clinicians, and those who disclose to researchers but not to clinicians are at elevated short-term risk (27; see also 28). Concealment is therefore not an unobservable absence but a measurable clinician-versus-researcher discrepancy with demonstrated predictive validity—and, measured alongside alliance and engagement, it allows the dissociation at the heart of H1 to be observed directly: sustained engagement coexisting with withheld intent.

Boundary conditions: The account does not predict a universal effect. A serious rival hypothesis is that, for some acutely dysregulated patients, directive structure is experienced as containing rather than coercive; the theory therefore predicts stronger effects in post-acute settings than during acute crisis. Two further moderators are specified *a priori*: patients with prior experience of involuntary admission should show larger concealment-under-containment effects, because the anticipated consequences of disclosure are more concrete; and adolescents, for whom autonomy concerns may be especially salient, are predicted to show stronger effects than adults—a candidate partial explanation of the limited paediatric evidence (29).

A staged research program: The hypothesis is best approached in stages, each independently publishable: Stage (0) would involve the development and validation of an atheoretical coding system for clinician stance. Stage (1) would involve experimental analogue studies that hold safety-plan content constant while only the stance varies, allowing direct tests of disclosure, concealment, and urge intensity. Stage (2) would involve naturalistic observational studies using clinician-versus-researcher disclosure discrepancies as an index of concealment, while examining moderators such as prior involuntary treatment and developmental stage. Stage (3) would involve pragmatic clinical trials comparing standard and parts-informed safety planning in post-acute settings. Acute patients should not be randomized to a deliberately suppressive intervention.

Defeat conditions: A useful hypothesis specifies not only what would support it but what would undermine it. If concealment of intent rises and falls together with disengagement—that is, if there is no dissociation, and the patients who conceal are simply the patients who have withdrawn—then H1 collapses into the alliance account and is not discriminating. If autonomy-supportive and containment-oriented deliveries produce equivalent selective concealment once engagement is accounted for, H1 fails. If disclosure does not differ by stance at all when content and contact time are controlled, H2 fails. If observed effects disappear once contact time is covaried, the theory collapses into a simpler explanation: that more time, not stance, produced the result. These outcomes would not undermine the value of collaborative care; they would undermine the specific claim advanced here—that manager enactment and protective concealment are the mechanisms through which stance exerts its effects. The discriminating test is whether containment-oriented delivery produces selective concealment of suicidal intent despite preserved engagement. If it does not, the enactment hypothesis should be rejected.

## 9 Objections and limitations

### 9.1 Objections

#### 9.1.1 “Honoring the suicidal part colludes with suicide”

The clinician validates the part’s pain and its protective intention but never its method. This is the same distinction CAMS draws when it targets and shifts drivers rather than endorsing them. The mechanism runs the other way: a firefighter that feels understood, and an exile that is witnessed and unburdened, lose the need to act. Suppression colludes with the polarity that drives the crisis; understanding dissolves it.

#### 9.1.2 “This is fine for outpatient therapy but not for an acute crisis”

Correct, and this is accommodated by the phase distinction. What is portable to the acute setting is the stance; the depth work is reserved for when risk is contained.

#### 9.1.3 “The evidence is not there”

Conceded, without flinching: IFS has no trial-grade evidence base for suicidality; the relevant literature is conceptual and clinical (6, 9), and the broader IFS evidence base, while promising, is limited (30). The argument is consequently modest in form: not “use an unproven therapy instead of a proven one,” but “carry an IFS-informed stance into CAMS and SPI, both of which have an evidence base, and which this stance plausibly enhances along exactly the dimensions—engagement, disclosure, and alliance—that the evidence identifies as mechanisms.” This is a hypothesis-generating position and should be read as one.

#### 9.1.4 “This is just DBT/Aeschi/CAMS in new words”

“This is just DBT/Aeschi/CAMS in new words.” The functional reading of suicidality is shared and attributed (Claim A). What is not shared is the relational-versus-behavioral ontology of the impulse and, decisively, the analysis of the clinician’s own procedure as a part-driven enactment—a move unavailable within a behavioral, narrative, or theoretically agnostic framework (Section 4).

### 9.2 Limitations

#### 9.2.1 The metaphysical commitment

It is worth stating openly that IFS makes a substantive and debatable claim: the existence of a Self that is undamaged, ever-present, and not itself a part. This aligns more closely with certain contemplative and depth-psychological traditions than with mainstream cognitive science, and clinicians should adopt it knowing its implications. The parallel to the “inner healing intelligence” invoked in psychedelic-assisted therapy is striking and worth pursuing elsewhere. However, the central argument of this paper does not depend on this claim. Whether “parts” are taken as literal subpersonalities or as a useful heuristic for organizing conflicting motivational states, and whether “Self” names an ontological entity or simply a regulated, non-defended stance the clinician can adopt, the enactment mechanism remains unchanged: a containment-oriented approach still recruits the clinician into the suppressive pole of the patient’s motivational conflict, and the concealment prediction (H1) still follows. Readers skeptical of IFS metaphysics can therefore interpret the entire account in a deflationary, as-if manner without losing any of its empirical content; the parts language earns its place by being clinically generative and by naming the role into which the clinician is recruited, not by requiring assent to a specific model of the psyche.

#### 9.2.2 Cultural generalizability

The account is developed within an individualized, Western therapeutic framework, and its unit of analysis is the dyadic encounter—a real limitation. In Australia, Aboriginal and Torres Strait Islander suicide is shaped by collective, intergenerational, and colonial determinants—dispossession, racism, and the disruption of social and cultural continuity—that an intrapsychic model does not capture and must not be taken to displace. Whether the relational emphasis of a Self-led stance maps usefully onto collective and kinship-based understandings of personhood, or instead imports an individualism that is ill-suited to them, is an empirical and ethical question to be answered through co-design with Aboriginal and Torres Strait Islander communities and practitioners, not assumed. The encounter-level mechanism is offered as potentially complementary to, never a substitute for, the social and cultural determinants of suicide and community-led prevention.

#### 9.2.3 Medico-legal coexistence

An IFS-informed stance does not exempt the clinician from documentation, risk frameworks, or duty of care; it operates within them. A function-decoding formulation is generally a stronger medico-legal artefact than a bare risk rating, because it provides evidence of clinical reasoning. The stance changes how the mandated work is done not whether it is done.

## 10 Discussion and conclusion

Critics have identified potential limitations of detection-and-containment approaches at the level of systems, policy, and clinical culture. The present paper has proposed an encounter-level mechanism through which those limitations may emerge within the clinical relationship itself.

The central proposal is straightforward. If suicidality is understood, at least in part, as a protective response to unbearable distress, then containment-oriented safety planning may recruit the clinician into the manager pole of the patient’s own manager–firefighter polarity. Under such conditions, the therapeutic encounter risks re-enacting the very conflict it seeks to resolve. The result may be not only polarization and rupture but protective concealment: a situation in which efforts to manage risk inadvertently increase incentives to withhold information about suicidal intent. The contribution is not the functional interpretation of suicidality, nor the critique of containment-oriented care—both well established—but the proposal that the clinical encounter itself may operate as an enactment of the patient’s polarity, together with the concealment mechanism through which suppression-oriented care may undermine its own aims.

The practical implications are correspondingly modest. Nothing in this account replaces safety planning, CAMS, means-safety counselling, existing duties of care, or the need for containment during acute crisis. The proposed change is one of stance rather than structure; the central claim is that stance is not a neutral background variable but an active ingredient of the intervention itself. For psychiatric education and supervision, the implication is less the adoption of a new protocol than the cultivation of a different form of attention: learning to recognize when safety planning begins to function primarily as control, when curiosity about function has given way to suppression, and when the clinician is being recruited into one pole of the patient’s conflict. Because enactment is, by definition, difficult to recognize from within, reflective supervision may be a more important training vehicle than additional procedural guidance.

The value of the present account ultimately rests not on its conceptual appeal but on its empirical fate. Alliance-based and autonomy-supportive theories already predict improved engagement, disclosure, and alliance; the discriminating question is whether containment-oriented delivery produces selective concealment of suicidal intent despite preserved engagement—the dissociation between sustained alliance and withheld intent that no rival account predicts. If it does not, the enactment hypothesis should be rejected. If it does, then one of the central assumptions of contemporary suicide-specific care may require reconsideration: whether the way we currently plan for safety may, under some conditions, suppress the very disclosure upon which that safety depends.

## Data Availability

The original contributions presented in the study are included in the article/supplementary material. Further inquiries can be directed to the corresponding author.
